# Long non-coding RNA TUG1 knockdown promotes autophagy and improves acute renal injury in ischemia-reperfusion-treated rats by binding to microRNA-29 to silence PTEN

**DOI:** 10.1186/s12882-021-02473-0

**Published:** 2021-08-24

**Authors:** Zhiquan Xu, Xiaoyan Huang, Qiuyu Lin, Wei Xiang

**Affiliations:** 1 Department of Nephrology, Rheumatology and Immunology, Hainan Women and Children’s Medical Center, 570300 Haikou, Hainan P.R. China; 2 Department of Genetics, Metabolism and Endocrinology, Hainan Women and Children’s Medical Center, 570206 Haikou, Hainan P.R. China; 3 Department of Respiratory, Hainan Maternal and Children’s Medical Center, 570000 Haikou, Hainan, P.R. China; 4 Department of Pediatrics, Hainan Maternal and Children’s Medical Center, Changbin Road, Xiuying District, Hainan 571199 Haikou, P.R. China

**Keywords:** Renal ischemia-reperfusion, Autophagy, Apoptosis, Long noncoding RNA TUG1, PTEN, mcroRNA-29

## Abstract

**Objective:**

Long noncoding RNA (lncRNA) taurine upregulated gene 1 (TUG1) is increased under the condition of ischemia. This study intended to identify the mechanism of TUG1 in renal ischemia-reperfusion (I/R).

**Methods:**

First, a rat model of acute renal injury induced by I/R was established, followed by the measurement of blood urea nitrogen (BUN), serum creatine (SCr), methylenedioxyphetamine (MDA) and superoxide dismutase (SOD) in the serum of rats. TUG1 was knocked down in I/R rats (ko-TUG1 group). Next, histological staining was used to evaluate the pathological damage and apoptosis of rat kidney. Western blot analysis was used to detect the levels of apoptosis- and autophagy-related proteins and transmission electron microscope was used to observe autophagosomes. Autophagy and apoptosis were evaluated after inhibition of the autophagy pathway using the inhibitor 3-MA. The targeting relation among TUG1, microRNA (miR)-29 and phosphatase and tensin homolog (PTEN) were validated. Lastly, the effects of TUG1 on biological behaviors of renal tubular cells were evaluated *in vitro*.

**Results:**

*In vivo*, the levels of BUN, SCr and MDA in the serum of I/R-treated rats were increased while SOD level and autophagosomes were reduced, tubule epithelial cells were necrotic, and TUG1 was upregulated in renal tissues of I/R-treated rats, which were all reversed in rats in the ko-TUG1 group. Autophagy inhibition (ko-TUG1 + 3-MA group) averted the protective effect of TUG1 knockdown on I/R-treated rats. TUG1 could competitively bind to miR-29 to promote PTEN expression. *In vitro*, silencing TUG1 (sh-TUG1 group) promoted viability and autophagy of renal tubular cells and inhibited apoptosis.

**Conclusions:**

LncRNA TUG can promote PTEN expression by competitively binding to miR-29 to promote autophagy and inhibited apoptosis, thus aggravating acute renal injury in I/R-treated rats.

**Supplementary Information:**

The online version contains supplementary material available at 10.1186/s12882-021-02473-0.

## Introduction

As a major cause of most cardiovascular diseases, ischemia/reperfusion (I/R) injury is recognized as a process when the blood supply returns to tissues after hypoxia, which causes ischemia and induces a cascade of events associated with oxidative damage and dysfunction [1]. Serious clinical manifestations such as acute heart failure, myocardial infarction, cerebral and gastrointestinal disorder, systemic inflammatory response and multiple organ dysfunctions are critical medical conditions of I/R injury [2]. Principally, clinical symptoms are often subtle at first, making it impossible to identify the exact onset time of ischemia; if diagnosed within 24 h after symptoms appear, the survival rate of acute ischemia is about 50 %, but this rate decreases to 30 % or even lower in case of delayed diagnosis [3]. Post-ischemic acute renal injury (AKI) is characterized by decreased glomerular filtration rate and high renal vascular resistance with endothelial activation and dysfunction, and it is a known process of critical importance followed by a reduction in microvascular blood flow mainly affecting the renal outer medulla [4]. I/R-induced AKI is a common and unavoidable phenomenon in kidney transplantation and may result in worsening or even loss of organ function [5]. Tubular cells are critical targets of I/R injury in renal transplantation [6]. If the biomarkers or targeted therapeutic interventions are to be explored, it is urgent to first comprehend the mechanism of renal tubular cells in response to I/R.

Emerging studies have implicated a fundamental role for non-coding RNAs, such as microRNAs (miRs) and long non-coding RNAs (lncRNAs) in acute I/R injury [7, 8]. LncRNA taurine upregulated gene 1 (TUG1) is essential for retinal development in the developing mouse eyes, and its abnormal expression is detected in tumorigenesis, either as a potential tumor suppressor or oncogene [9]. TUG1 has been identified to protect mouse livers against cold-induced liver damage in liver transplantation via inhibiting apoptosis and inflammation [10]. Essentially, it has been documented that hypoxia treatment significantly increases TUG1 expression and overexpression of TUG1 aggravates hypoxia‑induced injury in H9c2 cells [11]. Additionally, lncRNA TUG1 may function as a competing endogenous RNA (ceRNA) for miRs to induce cell damage, possibly providing a new target in cerebral I/R injury [12, 13]. In light of these references, we hypothesize there is an underlying ceRNA network involving TUG1 in renal I/R injury. Therefore, we carried out *in vivo* and *in vitro* experiments to figure out the protective mechanism of TUG1 in renal I/R injury.

## Materials and methods

### Ethics statement

All experimental protocols were recommended and approved by the Hainan Women and Children’s Medical Center (Approval number: SYXK(Qiong)2018-0015). The experimental process strictly followed the approved protocol. We made significant efforts to minimize the number of animals used and their suffering.

### Establishment of renal I/R model

Wistar rats (290 ± 10 g, 7–8 weeks old) provided by Laboratory Animal Center of Peking University Health Science Center were kept under a 12-h light/12-h dark cycle with rodent chow and tap water ad libitum in a temperature-controlled environment (20-22 °C) and 40–50 % humidity.

After adaptive feeding for 3 days, the rats were randomized to sham group (N = 12, rats were received surgical procedures without renal arterial clamping); I/R group (*N* = 12, rats underwent renal ischemia for 45 min followed by 24-h reperfusion); negative control (NC) group (*N* = 12, rats were received intravenous injection of short hairpin RNA (shRNA) NC vectors (5 × 10^7^ copies of the lentivirus with shRNA NC) for 15 min before renal artery clamping); knockdown TUG1 (ko-TUG1) group (*N* = 12, rats were injected with shRNA TUG1 (5 × 10^7^ copies of the lentivirus with shRNA TUG1) for 15 min after removing renal artery clamp), and ko-TUG1 + 3-MA group (N = 12, rats were injected with shRNA TUG1 (5 × 10^7^ copies of the lentivirus with shRNA TUG1) and 3-Methyladenine (3-MA) (30 mg/kg, Sigma-Aldrich, Merck KGaA, Darmstadt, Germany) [14] for 15 min after removing renal artery clamps). TUG1 shRNA and control shRNA were constructed into lentiviral vector PHY-LV-KD5.1 (ThermoFisher Scientific, Waltham, MA, USA) and then packaged into lentivirus particles [15].

Rats were anesthetized using 3 % sodium pentobarbital (i.p, 50 mg/kg). Then, the right kidney of each rat was removed, and the left renal artery was clamped for 45 min. After removing the clamp, the kidney was observed for 4–5 min to ensure the successful reperfusion. At 24 h post reperfusion, the left kidney of each rat was removed and rats were euthanized (Supplementary Figs. 1, 2). The serum of 12 rats in each group was applied for detection of serum indexes [blood urea nitrogen (BUN), blood creatinine (SCR), malondialdehyde (MDA) and superoxide dismutase (SOD)], and half of the kidney tissues of 12 rats were used for tissue homogenate, and another half of the kidney tissues of 12 rats were used for tissue section.

### Renal function assessment

Peripheral blood was put into the coagulation tube and centrifuged at 3000 r/min for 10 min. Then, 500 µL serum was taken to determine serum creatinine (SCr) and blood urea nitrogen (BUN) contents using an automatic biochemical analyzer (Roche Diagnostics, Indianapolis, Indiana, USA) with a kit from an AU2700 Analyzer (Olympus, Tokyo, Japan) [16].

### Detection of renal MDA and SOD contents

MDA content in rat kidneys was assessed by an MDA kit (Jiancheng Bioengineering Institute, Nanjing, Jiangsu, China) with the thiobarbituric acid method [17]. The renal SOD content was assessed by a SOD kit (Jiancheng) [18].

### Histological examination

After fixing in 10 % buffered formalin, kidney tissues were embedded in paraffin and sliced at 4-µm. Following deparaffinization and rehydration, the sections were stained with hematoxylin and eosin (HE) for quantitative evaluation of renal injury score. Furthermore, the histologic score was estimated with two external pathologists based on following criteria: tubular dilatation, cast deposition, brush border loss and necrosis in 25 arbitrarily selected non-overlapping fields. Pathological scoring is between 0 and 5 points based on the percentage of injury area: 0, no damage; 1, injury area < 10 %; 2, 10 % < injury area < 25 %; 3, 25 % < injury area < 50 %; 4, 50 % < injury area < 75 %; 5, injury area > 75 %.

### TUNEL assay

TUNEL assay was conducted with an In situ cell death detection fluorescein kit (Roche Diagnostics). Paraffined sections were deparaffinized, sliced at 4-µm, treated with 20 mg/mL proteinase K and 3 % hydrogen peroxide. Subsequently, the samples were treated with nucleotides and TdT enzyme at 37 °C for 1 h, followed by 30-min treatment with converter conjugated-horseradish peroxidase at 37 °C. Under a fluorescence microscope (Carl Zeiss, Jena, Germany), the cells with green stained nuclei were regarded as TUNEL-positive and calculated as the percentage of total cells. TUNEL-positive cells were calculated in 10 arbitrarily chosen fields (× 200) in a blind manner.

### Transmission electron microscopy (TEM)

The 1 mm^3^ renal samples were fixed with 2.5 % glutaraldehyde at 4 °C overnight, and treated with osmium tetroxide, embedded and sliced. The TEM (Hitachi, Tokyo, Japan) was used to observe the autophagosome ultrastructure [19].

### Immunofluorescence

The paraffin-sections were cut into 6–8 μm and incubated with light chain 3 (LC3) primary antibody (ab48394, Abcam) for 2 h. Then, the sections were incubated with goat anti-rat secondary antibody (ab150077, Abcam) combined with Alexa fluor® 488 at 37 °C for 30 min. The images were observed under a fluorescence microscope.

### Cell culture and grouping

TCMK-1 cells purchased from ATCC (CCL-139™) were cultured in Dulbecco modified Eagle medium with 10 % heat-inactivated fetal bovine serum (FBS, both from HyClone, Logan, UT, USA) at 37 °C with 5 % CO_2_. TCMK-1 cells were cultured in Forma Series II Water Jacketed CO_2_ incubator (ThermoFisher) with 94 % N_2_ and 5 % CO_2_ to keep 1 % oxygen concentration for hypoxia treatment. After that, cells were transferred to normoxic chamber (21 % O_2,_ 74 % N_2_, and 5 % CO_2_) during reoxygenation incubation [20].

Cells were allocated into blank group (cultured in the normoxic chamber at indicated time points), hypoxia/reoxygenation (H/R) group (treated with H/R), NC group (treated with shRNA NC, after H/R treatment), and sh-TUG1 group (treated with shRNA TUG1 after H/R treatment) [21] (Supplementary Fig. 3). All transfections were conducted with HiPerFect transfection reagent (QIAGEN, Valencia, CA, USA).

### Cell counting kit-8 (CCK-8)

Cell viability was assessed using a CCK-8 kit (Dojindo, Kumamoto, Japan). The absorbance at 450 nm was measured using a microplate reader (Bio-Rad). The percentage of living cells was calculated based on the ratio of absorbance of the H/R group to that of the blank group [22].

### Flow cytometry

Cells were seeded at 5 × 10^5^/mL cells/well and 1 mL/well into 6-well plates. After cell adherence for 24 h, cells were stimulated with hypoxia and H/R in the refreshed medium. Cells in each group were collected into flow tubes, and each tube was added with 5 uL fluorescein isothiocyanate-labeled Annexin-V buffer and 100 uL 1 × loading buffer, followed by 30-min incubation in conditions devoid of light. Cell apoptosis was detected using a flow cytometer (Beijing YourHope Medical Equipment Co., Ltd., Beijing, China). The apoptotic rate was expressed as the ratio of (premature aging cells + late aging cells) to the total number of cells.

### Monodansyl cadaverine (MDC) assay

A total of 1 × 10^5^ cells were seeded in 24-well plates and incubated at 37℃ for 24 h for attachment. Then, the cells were incubated with 0.5 mm MDC (Shanghai Yuduo Biotechnology Co., Ltd) at 37℃ for 10 min. The staining solution was discarded and the cells were rinsed with PBS three times. The slides were observed under a fluorescence microscope.

### RNA pull-down assay

TCMK-1 cells were delivered with biotinylated miR and collected after 48-h transfection. Next, the cell lysates were added with M-280 streptavidin magnetic beads (Invitrogen). Then, the bound RNAs were purified by TRIzol reagent for RT-qPCR [23].

### Dual-luciferase reporter gene assay

TUG1 and phosphatase and tensin homolog (PTEN) 3’untranslated region (3’UTR) sequences containing miR-29 binding site were synthesized, respectively, followed by construction of TUG1 and PTEN 3’UTR wild type (WT) and mutation (MUT) plasmids. Then, the constructed plasmids were delivered with NC and miR-29 plasmids into 293T cells (ATCC, Manassas, Virginia, USA). After 48-h transfection, the cells were lysed. Luciferase activity was detected by a luciferase detection kit (BioVision, SanFrancisco, CA, USA) and Glomax20/20 luminometer (Madison, WI, USA).

### Reverse transcription quantitative polymerase chain reaction (RT-qPCR)

The total RNA of renal tissues or tubular cells was extracted using TRIzol reagent (Invitrogen). After the determination of concentration and purity of RNA, cDNA was synthesized using a reverse transcription kit and amplified, and the expression of each primer in Table 1 was tested using a SYBR PCR Master Mix kit (Bio-Rad, Inc., Hercules, CA, USA), with U6 or β-actin serving as the reference. All primers were designed by Primer 3Plus website and synthesized by Genewiz Biotechnology (Suzhou, Jiangsu, China). The experiment was performed 3 times to calculate the average CT value, and the concentration of each sample was calculated based on the 2^−△CT^ method [24] to detect the relevant RNA level in cells and tissues.

**Table 1:** Primer sequences for RT-qPCR

Primer	Sequence (5’→3’)
TUG1	F: GCTATTGGTATGGCTGGCCT
	R: AAGGAGAGAAATGGACGCGG
CHCHD4P4	F: TGACCCCACCTCTTCTTTGG
	R:AGACATTAACTGGAACCGTCC
AU015836	F: GCCTCCCAGCCATTAGGTTT
	R: GCCACCGTGTAGAGGTCAAA
MALAT1	F: CAGCAGCAGACAGGATTCCA
	R: ATTGCCGACCTCACGGATTT
HIF1A-AS1	F: TGGATGCCCACATGCATTATGA
	R: AGCAAGGGCTGTTCCATGTT
PVT1	F: GGGGAATAACGCTGGTGGAA
	R: CCCATGGACATCCAAGCTGT
PTEN	F: GTGCAGATAATGACAAG
	R: GATTTGACGGCTCCTCT
β-actin	F: GTCATTCCAAATATGAGAGATGCGT
	R: GCTATCACCTCCCCTGTGTG
miR-29	F: TGCCAGGAGCTGGTGATTTCCT
	R: ACGGGCGTACAGAGGATCCCC
U6	F: AACGCTTCACGAATTTGCGT
	R: GGTGTACTCTTGGGGAACCAG

Note: *RT-qPCR* reverse transcription quantitative polymerase chain reaction; *TUG1* taurine upregulated gene 1; *PTEN* phosphatase and tensin homolog; *miR-29* microRNA-29; *F* forward; *R* reverse

### Western blot analysis

After homogenization in radio immunoprecipitation assay lysis buffer (Beyotime, Shanghai, China), the renal tissues were centrifuged (12,000 g, 4 °C, 10 min). Then, the proteins were quantified by a bicinchoninicacid kit (ThermoFisher), and then subjected to 15 % sodium dodecyl sulfate-polyacrylamide gel electrophoresis (SDS-PAGE) and transferred onto nitrocellulose membranes. Membrane blockade was conducted for 2 h with 5 % non-fat dry milk in tris-buffered saline-tween (TBST) at 37 °C, followed by overnight incubation with corresponding primary antibodies (Table 2) at 4 °C. After TBST rinses, the membranes underwent 1-h incubation with horseradish-peroxidase conjugated secondary antibody (ab205718, dilution ratio of 1:2000, Abcam). After TBST rinses, the protein bands were visualized using enhanced chemiluminescence and Odyssey Infrared Imaging System (LI-COR Biosciences, Lincoln, NE, USA). Target protein/glyceraldehyde-3-phosphate dehydrogenase (GAPDH) was used as relative protein expression.

**Table. 2:** Antibodies used in experiment

Antibody	Information	Dilution rate
Bax	ab32503, Abcam	1/1000
Bcl-2	ab182858, Abcam	1/2000
p62	ab91526, Abcam	2 µg/mL
Beclin-1	ab207612, Abcam	1/2000
LC3II	ab48394, Abcam	2 µg/mL
LC3I	ab192890, Abcam	1/2000
PTEN	ab32199, Abcam	1/10,000
GAPDH	ab181602, Abcam	1/10,000

Note: Bcl-2, B-cell lymphoma-2; Bax, Bcl-2-associated X; LC3, light chain 3; PTEN, phosphatase and tensin homolog; GAPDH, glyceraldehyde-3-phosphate dehydrogenase

### Statistical analysis

Statistical analysis was conducted using the SPSS 21.0 (IBM Corp. Armonk, NY, USA). All the data were in normality distribution evaluated with the Kolmogorov-Smirnov test. Measurement data were expressed as mean ± standard deviation. The *t* test was used to process comparisons between two groups, while one-way analysis of variance (ANOVA) was used to analyze comparisons among multiple groups, and Tukey’s multiple comparisons test was used for post-hoc test. The *p* value was obtained using a two-tailed test and a value of *p* < 0.05 indicated a statistical difference.

## Results

### TUG1 is upregulated in renal I/R-treated rats

**Fig. 1:**
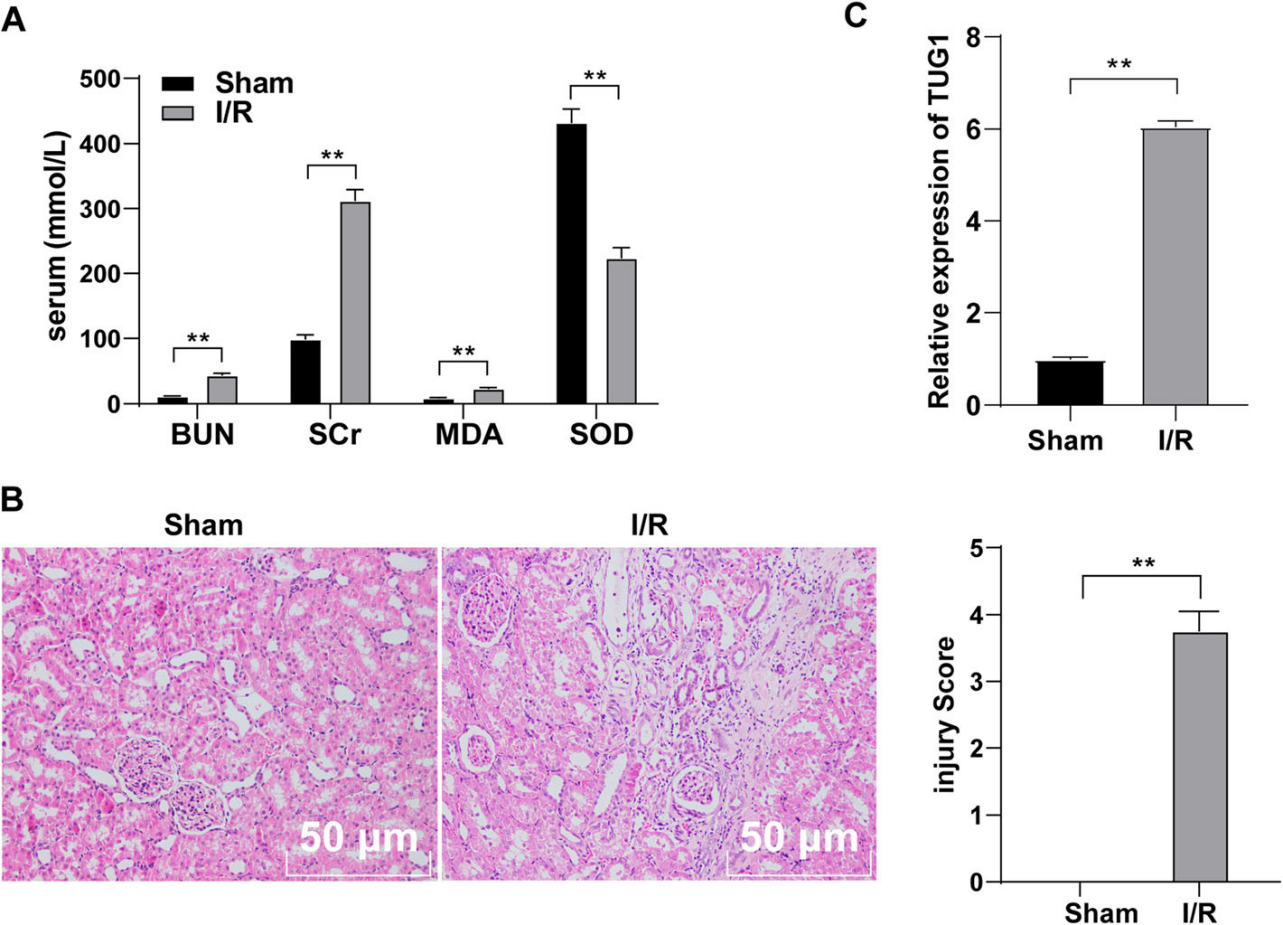
TUG1 is upregulated in renal I/R-treated rats. **A**, levels of BUN and SCr in serum of rats were detected using automatic biochemical instrument, and levels of MDA and SOD in the serum of rats were detected using corresponding kits (*N* = 12); **B**, HE staining evaluated the pathological damage of renal tissues (× 200) (*N* = 12); C, RT-qPCR detected the TUG1 expression in sham-operated rats and I/R-treated rats (*N* = 12). Data were analyzed using the *t* test. **p < 0.01

The levels of serum BUN, SCr, MDA and SOD were measured in the sham group and I/R group. Compared with the sham-operated rats, the levels of BUN, SCr and MDA in the serum of I/R-treated rats were elevated, and the level of SOD was decreased significantly (all *p* < 0.01) (Fig. 1 A). Then, HE staining was used to evaluate the pathological damage of renal tissues. The results showed clear and complete structure of renal tubules in sham-operated rats, while the renal tissues in I/R-treated rats were seriously damaged (Fig. 1B), indicating the successful establishment of I/R rat model.

We detected the expression of TUG1 in the renal tissues of the two groups by RT-qPCR. Compared with the sham-operated rats, TUG1 was significantly upregulated in I/R-treated rats (*p* < 0.01) (Fig. 1 C). These results indicated that the expression of lncRNA TUG1 was significantly elevated in rats with acute I/R injury.

### TUG1 knockdown improves renal IR injury and inhibits cell apoptosis

**Fig. 2:**
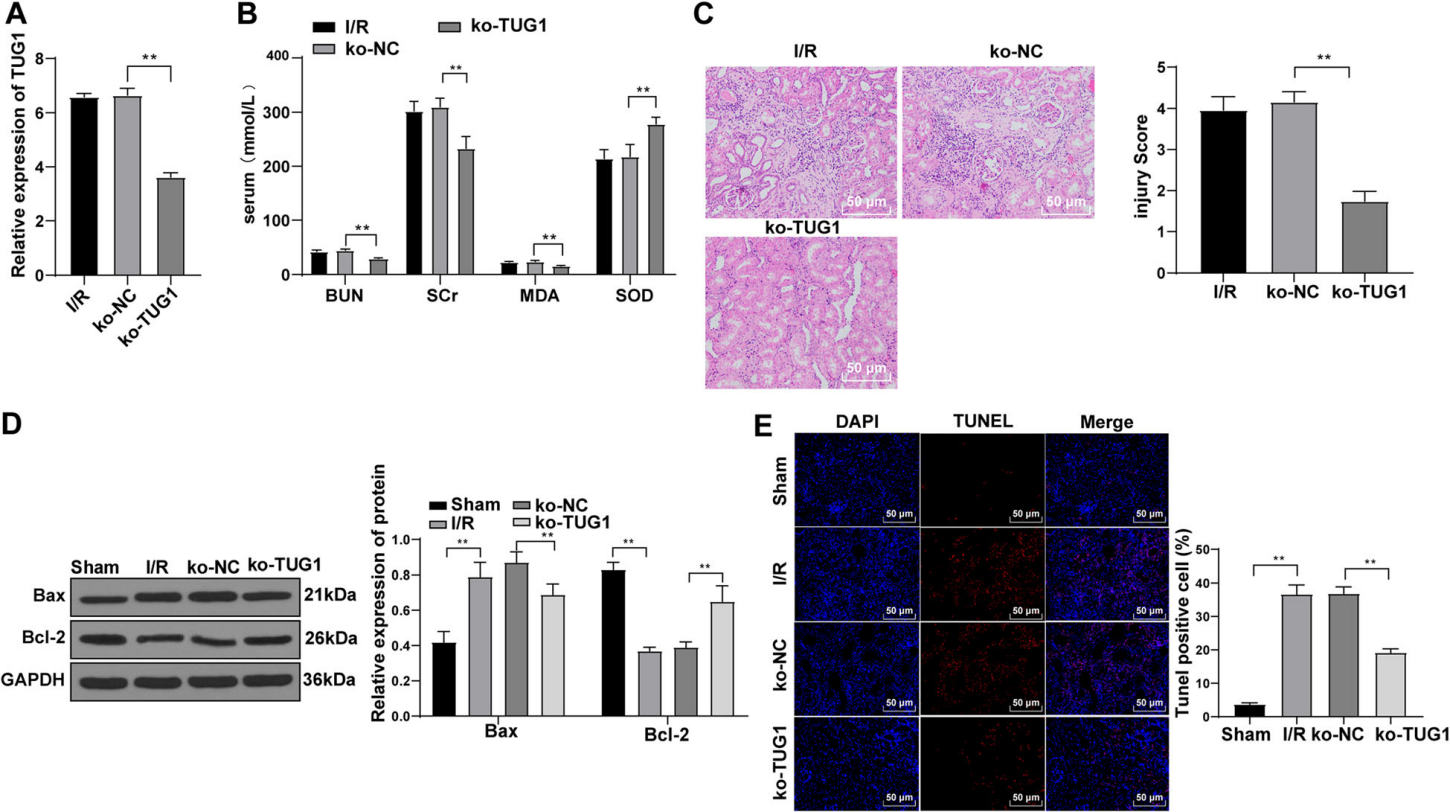
TUG1 knockdown improves renal IR injury and inhibits cell apoptosis. **A**, The effect of TUG1 knockdown on the expression of TUG1 was detected using RT-qPCR; **B**, levels of BUN and SCr in the serum of rats were detected using automatic biochemical instrument, and levels of MDA and SOD in the serum of rats were detected using corresponding kits (*N* = 12); **C**, HE staining evaluated the pathological damage of renal tissues (× 200) (*N* = 12); **D**, western blot analysis detected the levels of apoptotic proteins (*N* = 12); E, TUNEL assay measured the TUNEL-positive cells in renal tissues of rats (*N* = 12). Data were analyzed by one-way ANOVA, followed by Tukey’s multiple comparisons test. **p < 0.01, ***p < 0.01

We injected the shRNA TUG1 into I/R-treated rats, and verified the efficiency of TUG1 knockdown (Fig. 2 A). Then, the levels of BUN, SCr, MDA and SOD in the serum of rats were measured. Compared with the NC group, the serum levels of BUN, SCr and MDA in the ko-TUG1 group were reduced, while SOD level was considerably elevated (all *p* < 0.01) (Fig. 2B). Next, pathological morphology of the rats was evaluated using HE staining, which revealed that compared with the ko-NC group, the pathological morphology was improved after the acute renal I/R injury in the ko-TUG1 group (Fig. 2 C). After that, Western blot analysis and TUNEL staining were performed to detect the apoptosis of renal cells. The results showed that the Bax expression was reduced and Bcl-2 was enhanced, and TUNEL-positive cells in the ko-TUG1 group were notably reduced (all *p* < 0.01) (Fig. 2DE). These results suggest that silencing lncRNA TUG1 can improve acute renal injury induced by I/R.

### TUG1 knockdown promotes autophagy in rats with AKI induced by I/R

**Fig. 3:**
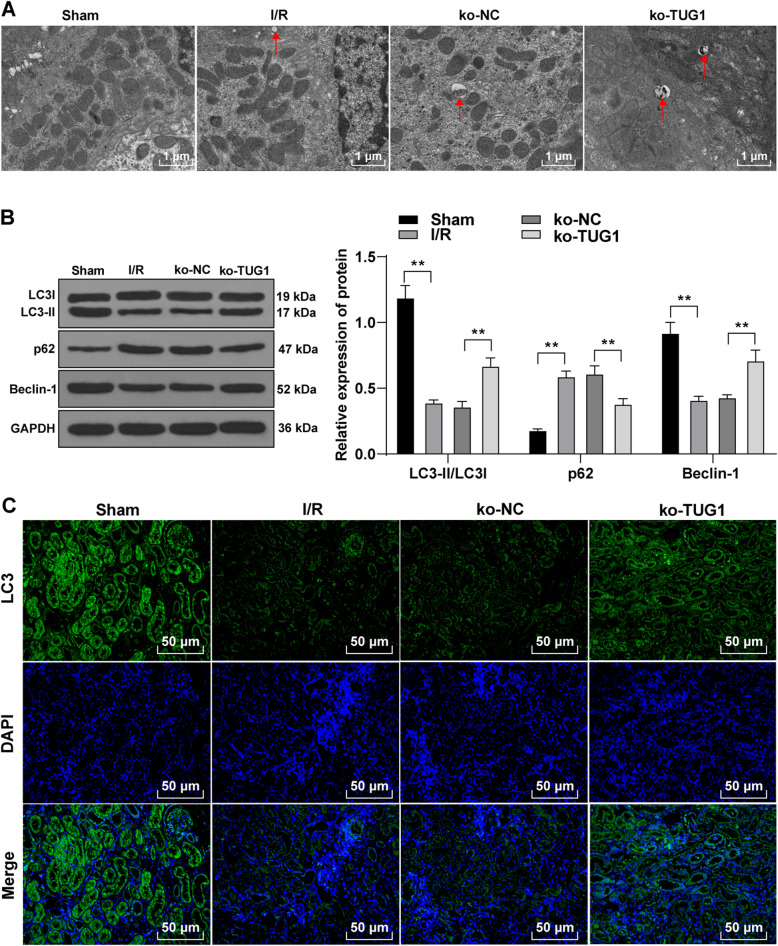
TUG1 knockdown promotes autophagy in rats with acute renal injury induced by I/R. **A**, the number of autophagosomes (× 5000) (*N* = 12) was observed by transmission electron microscope; **B**, western blot analysis detected the levels of autophagy-related proteins (*N* = 12); **C**, Immunofluorescence staining detected the fluorescence intensity of LC3 (× 400) (*N *= 12). Data in were analyzed by one-way ANOVA, followed by Tukey’s multiple comparisons test. **p < 0.01

Then, we explored the effect of TUG1 on autophagy in AKI induced by I/R. The results of TEM observation showed that compared with the sham group, the autophagosomes in renal tissue of rats in the I/R group were significantly reduced, but were notably increased after knocking down TUG1 (Fig. 3 A). Additionally, Western bot analysis was used to detect the levels of autophagy-related proteins. Relative to the sham group, LC3-II/LC3I and Beclin-1 levels in the I/R group were clearly decreased while the level of p62 was elevated. After knocking down TUG1, the autophagy-related proteins showed opposite trends (all *p* < 0.01) (Fig. 3BC). Immunofluorescence staining showed that relative to the I/R group, the fluorescence intensity of LC3 was increased after knocking down TUG1. These results suggest that knockdown of lncRNA TUG1 promotes autophagy in renal tissue of rats with acute renal I/R injury.

### Inhibition of autophagy attenuates the protective effect of TUG1 knockdown on rats with AKI induced by I/R

**Fig. 4:**
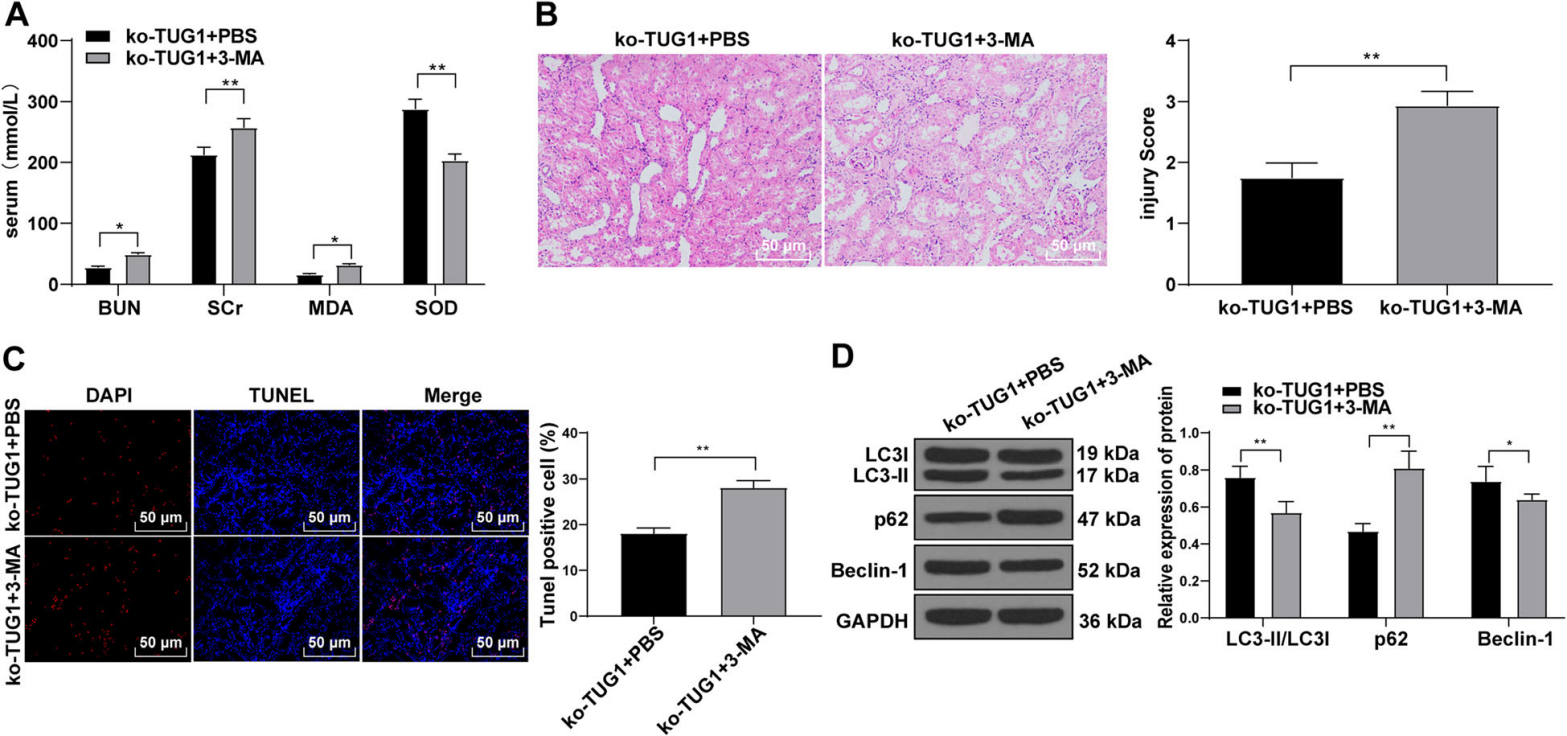
Inhibition of autophagy pathway attenuates the protective effect of TUG1 knockdown on rats with acute renal injury induced by I/R. **A**, levels of BUN and SCr in the serum of rats were detected using automatic biochemical instrument, and levels of MDA and SOD in the serum of rats were detected using corresponding kits (*N* = 12); **B**, HE staining evaluated the pathological damage of renal tissues (× 200) (*N* = 12); **C**, TUNEL assay measured the TUNEL-positive cells in renal tissues of rats (× 200) (*N* = 12); **D**, western blot analysis detected the levels of autophagy-related proteins (*N* = 12). Data were analyzed by the *t* test. *p < 0.05, **p < 0.01

To further identify the protective effect of TUG1 on rats with AKI induced by I/R through autophagy, a functional rescue experiment was carried out by setting up a combination group of autophagy pathway inhibitor (3-mA) and knockdown TUG1. Compared with the ko-TUG1 + PBS group, the serum levels of BUN, SCr and MDA in rats in the ko-TUG1 + 3-mA group were significantly increased, while SOD was reduced (all *p* < 0.01) (Fig. 4 A). HE staining revealed that the pathological morphology was improved after adding 3-mA (*p* < 0.01) (Fig. 4B). The TUNEL-positive cells were increased, LC3-II/LC3I and Beclin-1 level were reduced, and p62 level was increased in the ko-TUG1 + 3-MA group (all *p* < 0.01) (Fig. 4 A-D). Briefly, inhibition of autophagy annulled the protective effect of TUG1 knockdown on rats with AKI induced by I/R.

### TUG1 competitively binds to miR-29 to promote PTEN expression

**Fig. 5:**
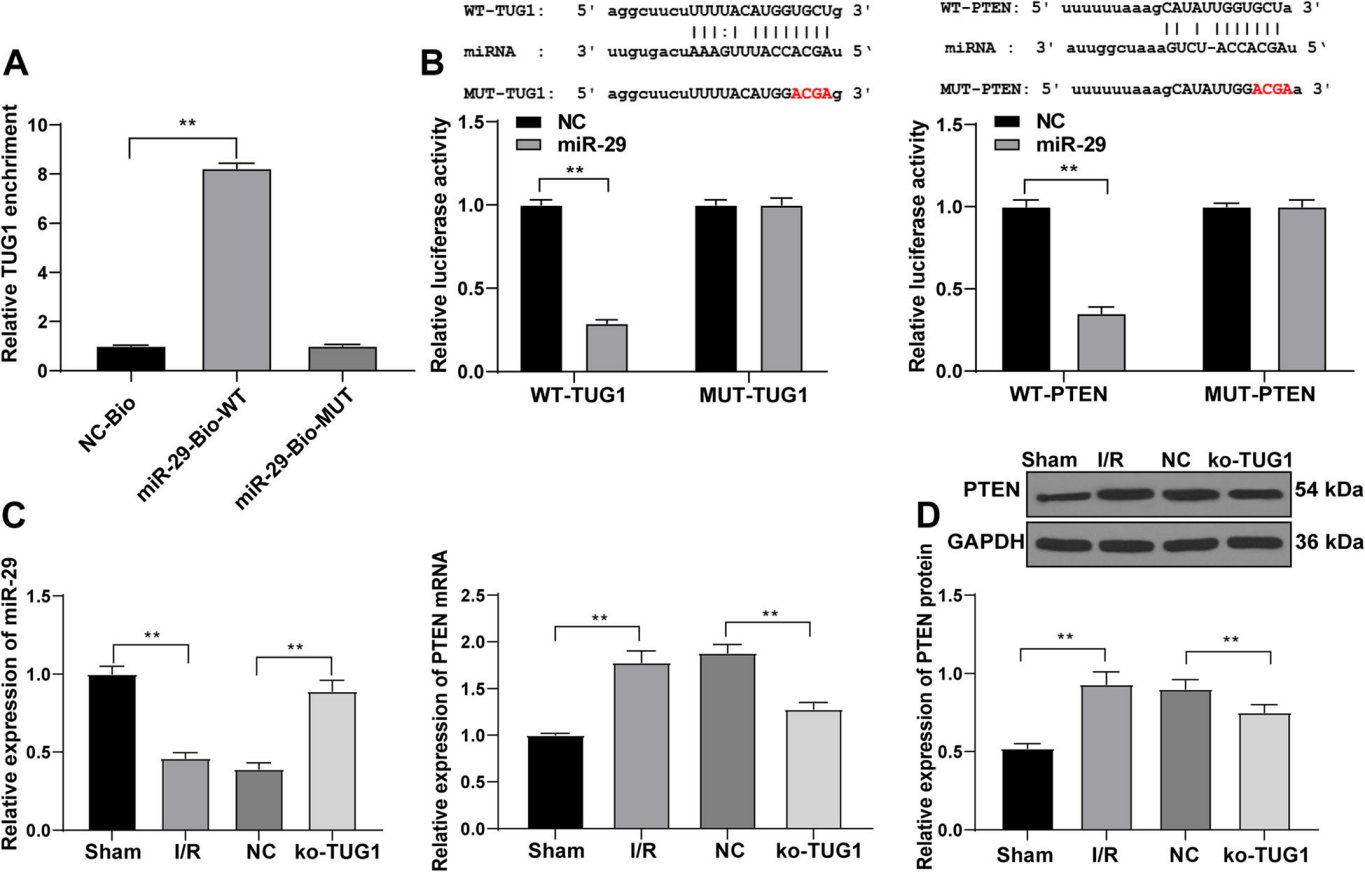
TUG1 competitively binds to miR-29 to promote PTEN expression. **A**, pull-down assay confirm a binding site between TUG1 and miR-29; **B**, dual-luciferase reporter gene assay verified a binding site between TUG1 and miR-29, and a binding site between PTEN and miR-29; **C**, RT-qPCR detected the expression of PTEN and miR-29 in kidney tissues; **D**, western blot analysis detected protein level of PTEN. The cell experiment was done 3 times. Data were analyzed by one-way ANOVA, followed by Tukey’s multiple comparisons test. **p < 0.01

To find out whether there is a ceRNA network involving TUG1 in I/R injury, a targeting relationship between TUG1 and multiple miRs has been identified based on our prediction through bioinformatics website (http://starbase.sysu.edu.cn/index.php). miR-29a overexpression has been reported to protect against I/R injury [25]. Expectedly, a binding site between TUG1 and miR-29 was identified through pull-down assay (Fig. 5 A). Then, we also predicted that there are multiple target genes of miR-29a, including PTEN. At the same time, PTEN can affect the process of AKI-induced by I/R [26–28]. The dual-luciferase reporter gene assay further verified the binding relationship between TUG1 and miR-29, and between miR-29 and PTEN (Fig. 5B). Compared with the sham group, miR-29 expression pattern in the I/R group was reduced while PTEN levels were elevated; compared with the NC group, the miR-29 expression pattern in the ko-TUG1 group was elevated partially, while PTEN levels were downregulated (all *p* < 0.05) (Fig. 5CD).

### Silencing TUG1 promotes viability and autophagy of TCMK-1 cells and inhibits apoptosis

**Fig. 6:**
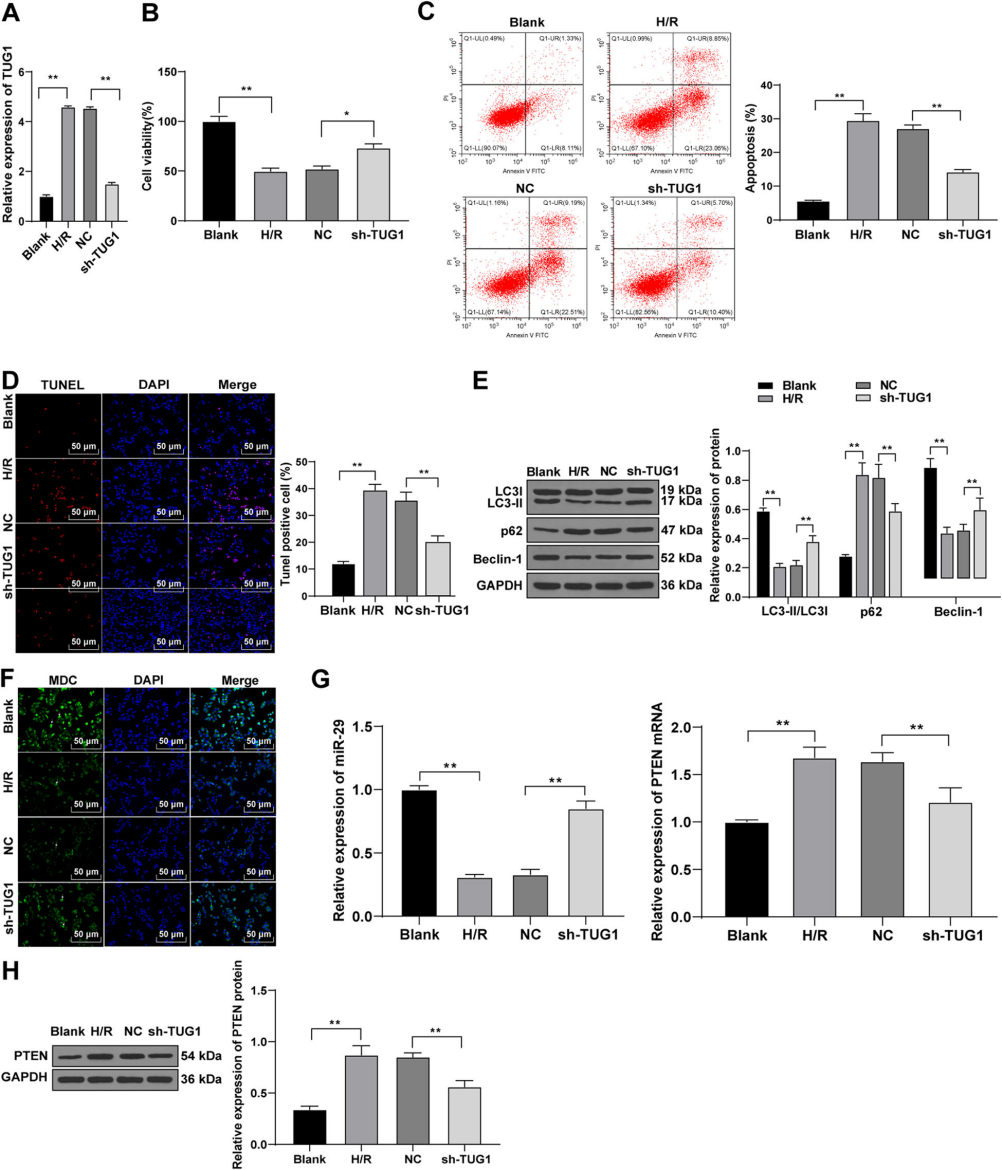
Low expression of TUG1 promotes viability and autophagy of renal tubular cells and inhibits apoptosis. **A**, The effect of shRNA TUG1 on the expression of TUG1 was detected using RT-qPCR; **B**, CCK-8 method detected cell viability in each group; **C**, flow cytometry detected cell apoptosis in each group; **D**, TUNEL assay measured the TUNEL-positive cells in renal tissues of rats in each group (× 200); **E**, western blot analysis detected the levels of autophagy-related proteins; **F**, MDC assay detected autophagy; **G**, RT-qPCR detected the expression of PTEN and miR-29 in kidney tissues; **H**, western blot analysis detected protein level of PTEN. Data were analyzed by one-way ANOVA, followed by Tukey’s multiple comparisons test. **p < 0.01

To verify the effect of TUG1 *in vitro*, we simulated the I/R injury of renal tubular cells TCMK-1 by H/R, and knocked down TUG1 expression. The efficiency of TUG1 knockdown was verified using RT-qPCR (Fig. 6 A). TCMK-1 cell viability in each group was measured using CCK-8 method. The result showed that the activity of TCMK-1 cells in the H/R group was lower than that in the blank group, and the activity of TCMK-1 cells in the sh-TUG1 group was higher than that in the NC group (all *p* < 0.01) (Fig. 6B). Flow cytometry and TUNEL staining elicited that compared with the blank group, the apoptosis rate of TCMK-1 cells in the H/R group was significantly enhanced; compared with the NC group, the apoptosis rate in the sh-TUG1 group was decreased (Fig. 6CD). Additionally, Western blot analysis and immunofluorescence staining revealed that compared with the blank group, the H/R group presented inhibited autophagy of TCMK-1 cells; compared with the NC group, the sh-TUG1 group presented promoted autophagy of TCMK-1 cells (all *p* < 0.01) (Fig. 6E). The result of MDC assay was consistent (Fig. 6 F). Furthermore, the levels of miR-29 and PTEN were detected using RT-qPCR and western blot analysis. Compared with the blank group, miR-29 expression pattern in TCMK-1 cells was reduced, and PTEN levels in the H/R group were significantly elevated; compared with the NC group, miR-29 expression pattern in TCMK-1 cells was upregulated, and PTEN levels in the sh-TUG1 group were significantly decreased (Fig. 6G/H) (all *p* < 0.01).

## Discussion

**Fig. 7:**
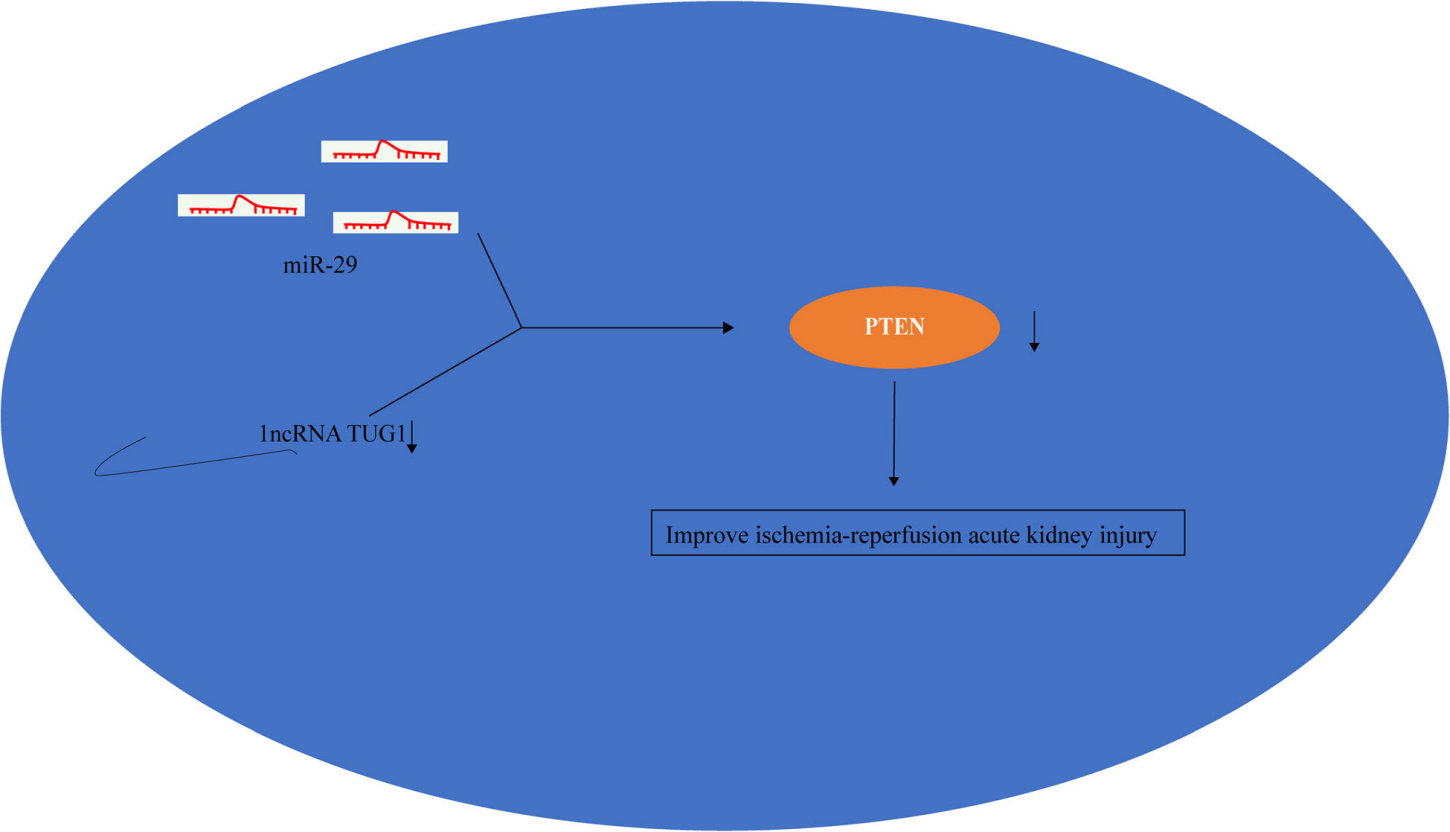
Mechanism diagram. Knockdown of lncRNA TUG1 inhibits the expression of PTEN by competitively binding to miR-29, thus improving the acute renal injury induced by ischemia-reperfusion

Renal ischemia, the most common cause of AKI is related to adverse outcomes and high mortality; it is estimated that AKI occurs in about 1 of 5 hospitalizations and is associated with a more than 4-fold increased likelihood of death [29, 30]. Therefore, it is of prime urgency and importance to search for effective therapeutic approaches for I/R injury, especially for renal I/R injury. Overexpression of lncRNA TUG1 has been identified in oxygen-glucose deprivation/reperfusion (OGD/R)-induced myocardial HL-1 cells [31]. This study highlighted that TUG1 knockdown could promote autophagy and improve AKI in I/R-treated rats by binding to miR-29 to silence PTEN expression (Fig. 7).

It has been reported that TUG1 is upregulated in the brain of rats with middle cerebral artery occlusion (MCAO) and OGD/R-treated SH-SY5Y cells, indicating the therapeutic potential of TUG1 in I/R [32]. In this study, TUG1 was significantly upregulated in I/R-treated rats. We speculated that lncRNA TUG1 had an effect on AKI- induced by I/R. To confirm this conjecture, we silenced TUG1 expression in I/R-treated rats to evaluate its effect. SOD could limit oxidative stress and renal I/R injury and is regarded as the most relevant molecule against I/R-induced changes [33]. Our results elicited that the levels of BUN, SCr and MDA in the serum of I/R-treated rats were increased, and the level of SOD was found to be decreased significantly, which were reversed after knocking down TUG1 in I/R treated rats. The urine volume, BUN, SCr and MDA concentrations, LC3II/LC3I, and autophagosomes were significantly elevated 24 h after renal I/R, while p62 and SOD concentrations were decreased [18]. Significantly enhanced myocardial SOD activity and reduced MDA level are beneficial for I/R-treated rats [34]. Thus, our study demonstrated that TUG1 knockdown may be beneficial for the treatment of renal I/R injury.

Autophagy is responsible for damaged organelles, and provides energy for cell renewal and internal environment stability, and loss of autophagy is associated with I/R injury [35]. LC3 and Beclin-1 are specific markers to monitor autophagy, and the amount of LC3-II is closely correlated with autophagosomes [36, 37]. After knocking down TUG1, the autophagosomes and levels of LC3-II/LC3I and Beclin-1 in renal tissues of rats were notably increased while level of p62 was reduced. A study elucidated that the upregulation of autophagy was associated with a 45 % reduction in infarct size [38]. TUG1 knockdown reduces the infarction area and cell apoptosis in MCAO model mice, thus effectively protecting against brain I/R injury [12]. Altogether, knockdown TUG1 could alleviate renal I/R injury by promoting autophagy.

I/R-induced TUG1 binds to miR-132-3p to activate histone deacetylase 3 and then provokes intracellular reactive oxygen species accumulation, and worsens the injury of acute myocardial infarction [39]. In our study, bioinformatics website predicted that lncRNA TUG1 has a targeted relationship with multiple miRNAs. Among them, increasing level of miR-29a can protect against I/R injury [25]. A close association between TUG1 and miR-29b was verified in the inhibition of apoptosis and inflammation in lipopolysaccharide-treated H9c2 cells [40]. Furthermore, we discovered that TUG1 competitively bound to miR-29 to promote PTEN expression. The expression of PTEN was upregulated in renal I/R injury [25]. The directing targeting relationship between miR-29a and PTEN was reported previously in osteosarcoma cells [41]. miR-29a mimic protects against cell injury and mitochondrial dysfunction after ischemia-like stresses *in vitro*, and increasing miR-29a expression might be a novel option for protection against I/R injury [26]. Briefly, TUG1 could competitively bind to miR-29 to promote PTEN in I/R injury.

Apoptosis is a process of programmed cell death that is activated under hypoxic stress in ischemic injury and during the production of ROS in reperfusion injury [42]. Then, the effect of lncRNA TUG1 on renal I/R injury was confirmed *in vitro*. In the present study, proliferation and autophagy of TCMK-1 cells were promoted and apoptosis was inhibited upon silencing TUG1. Activated autophagy notably reduces renal tissue damage and tubular cell apoptosis in I/R and H/R models [14]. Similarly, TUG1 was upregulated in ischemic heart and cardiomyocytes while knockdown of TUG1 inhibited cardiomyocyte apoptosis and markedly ameliorated impaired cardiac function of myocardial infarction mice by upregulating miR-9 expression [43]. Altogether, silencing TUG1 alleviated H/R injury *in vitro*.

In summary, TUG1 knockdown could promote autophagy and improves AKI in I/R-treated rats by binding to miR-29 to silence PTEN. However, the studies conducted on animal models to date are worthy of further research, because many underlying mechanisms are still unknown and need to be clarified. Additional clinical trials are warranted to justify this approach for lncRNA TUG1 target therapy in the future.

## Supplementary Information

Additional file 1: **Supplementary Figures S1, S2 and S3** Flow chart of cell experiments and animal experiments

## Data Availability

The data that support the findings of this study are available from the corresponding author upon reasonable request.

## Declarations

### Statements

All methods were carried out in accordance with the Hainan Women and Children’s Medical Center whose guidelines have been followed.

The study was carried out in compliance with the ARRIVE guidelines.

## References

[1] Raghay K, Akki R, Bensaid D, Errami M (2020). Ghrelin as an anti-inflammatory and protective agent in ischemia/reperfusion injury. Peptides.

[2] Wu MY, Yiang GT, Liao WT, Tsai AP, Cheng YL, Cheng PW, Li CY (2018). Li CJ. Current Mechanistic Concepts in Ischemia and Reperfusion Injury. Cell Physiol Biochem.

[3] Kalogeris T, Baines CP, Krenz M, Korthuis RJ (2012). Cell biology of ischemia/reperfusion injury. Int Rev Cell Mol Biol.

[4] Rodriguez F, Bonacasa B, Fenoy FJ, Salom MG (2013). Reactive oxygen and nitrogen species in the renal ischemia/reperfusion injury. Curr Pharm Des.

[5] Lorenzen JM (2015). Vascular and circulating microRNAs in renal ischaemia-reperfusion injury. J Physiol.

[6] Barin-Le Guellec C, Largeau B, Bon D, Marquet P, Hauet T (2018). Ischemia/reperfusion-associated tubular cells injury in renal transplantation: Can metabolomics inform about mechanisms and help identify new therapeutic targets?. Pharmacol Res.

[7] Ong SB, Katwadi K, Kwek XY, Ismail NI, Chinda K, Ong SG, Hausenloy DJ (2018). Non-coding RNAs as therapeutic targets for preventing myocardial ischemia-reperfusion injury. Expert Opin Ther Targets.

[8] Yang J, Chen M, Cao RY, Li Q, Zhu F (2018). The Role of Circular RNAs in Cerebral Ischemic Diseases: Ischemic Stroke and Cerebral Ischemia/Reperfusion Injury. Adv Exp Med Biol.

[9] Zhao XB, Ren GS (2016). LncRNA Taurine-Upregulated Gene 1 Promotes Cell Proliferation by Inhibiting MicroRNA-9 in MCF-7 Cells. J Breast Cancer.

[10] Su S, Liu J, He K, Zhang M, Feng C, Peng F, Li B, Xia X (2016). Overexpression of the long noncoding RNA TUG1 protects against cold-induced injury of mouse livers by inhibiting apoptosis and inflammation. FEBS J..

[11] Wu Z, Zhao S, Li C, Liu C (2018). LncRNA TUG1 serves an important role in hypoxia-induced myocardial cell injury by regulating the miR1455pBinp3 axis. Mol Med Rep.

[12] Shan W, Chen W, Zhao X, Pei A, Chen M, Yu Y, Zheng Y, Zhu S (2020). Long noncoding RNA TUG1 contributes to cerebral ischaemia/reperfusion injury by sponging mir-145 to up-regulate AQP4 expression. J Cell Mol Med.

[13] Xu Y, Deng W, Zhang W (2018). Long non-coding RNA TUG1 protects renal tubular epithelial cells against injury induced by lipopolysaccharide via regulating microRNA-223. Biomed Pharmacother.

[14] Guan X, Qian Y, Shen Y, Zhang L, Du Y, Dai H, Qian J, Yan Y (2015). Autophagy protects renal tubular cells against ischemia / reperfusion injury in a time-dependent manner. Cell Physiol Biochem.

[15] Zhang YL, Qiao SK, Wang RY, Guo XN (2018). NGAL attenuates renal ischemia/reperfusion injury through autophagy activation and apoptosis inhibition in rats. Chem Biol Interact.

[16] Qu Y, Sun Q, Song X, Jiang Y, Dong H, Zhao W, Li C (2020). Helix B surface peptide reduces sepsis-induced kidney injury via PI3K/Akt pathway. Nephrology (Carlton).

[17] Bian Y, Deng C, Li W, Lei Z, Li Y, Li X (2016). A Comparative Study on the Biological Characteristics of Human Adipose-Derived Stem Cells from Lipectomy and Liposuction. PLoS One.

[18] Ling Q, Yu X, Wang T, Wang SG, Ye ZQ, Liu JH (2017). Roles of the Exogenous H2S-Mediated SR-A Signaling Pathway in Renal Ischemia/ Reperfusion Injury in Regulating Endoplasmic Reticulum Stress-Induced Autophagy in a Rat Model. Cell Physiol Biochem.

[19] Chen GZ, Shan XY, Li XS, Tao HM (2018). Remote ischemic postconditioning protects the brain from focal ischemia/reperfusion injury by inhibiting autophagy through the mTOR/p70S6K pathway. Neurol Res.

[20] Yang Z, Zhuan B, Yan Y, Jiang S, Wang T (2016). Roles of different mitochondrial electron transport chain complexes in hypoxia-induced pulmonary vasoconstriction. Cell Biol Int.

[21] Zhang W, Li Y, Wang P (2018). Long non-coding RNA-ROR aggravates myocardial ischemia/reperfusion injury. Braz J Med Biol Res.

[22] Son M, Oh S, Choi CH, Park KY, Son KH,Byun K. Pyrogallol-Phloroglucinol-6,6-Bieckol from Ecklonia cava Attenuates Tubular Epithelial Cell (TCMK-1) Death in Hypoxia/Reoxygenation Injury. Mar Drugs. 2019;17(11):602. 10.3390/md17110602 PMC6891818 31652920

[23] Tsai MC, Manor O, Wan Y, Mosammaparast N, Wang JK, Lan F, Shi Y, Segal E (2010). Chang HY. Long noncoding RNA as modular scaffold of histone modification complexes. Science.

[24] Livak KJ, Schmittgen TD (2001). Analysis of relative gene expression data using real-time quantitative PCR and the 2(-Delta Delta C(T)) Method. Methods.

[25] Ouyang YB, Xu L, Lu Y, Sun X, Yue S, Xiong XX, Giffard RG (2013). Astrocyte-enriched miR-29a targets PUMA and reduces neuronal vulnerability to forebrain ischemia. Glia.

[26] Huang C, Chen Y, Lai B, Chen YX, Xu CY, Liu YF (2021). Overexpression of SP1 restores autophagy to alleviate acute renal injury induced by ischemia-reperfusion through the miR-205/PTEN/Akt pathway. J Inflamm (Lond).

[27] Sasaki K, Terker AS, Pan Y, Li Z, Cao S, Wang Y, Niu A, Wang S, Fan X, Zhang MZ, et al. Deletion of Myeloid Interferon Regulatory Factor 4 (Irf4) in Mouse Model Protects against Kidney Fibrosis after Ischemic Injury by Decreased Macrophage Recruitment and Activation. J Am Soc Nephrol. 2021;32(5):1037-1052. 10.1681/ASN.2020071010 PMC8259665 33619052

[28] Zhang Y, Wang J, Yang B, Qiao R, Li A, Guo H, Ding J, Li H, Ye H, Wu D (2020). Transfer of MicroRNA-216a-5p From Exosomes Secreted by Human Urine-Derived Stem Cells Reduces Renal Ischemia/Reperfusion Injury. Front Cell Dev Biol.

[29] Lambert E, Schlaich M (2017). The role of renal sympathetic nerves in ischemia reperfusion injury. Auton Neurosci.

[30] Wang HE, Muntner P, Chertow GM, Warnock DG (2012). Acute kidney injury and mortality in hospitalized patients. Am J Nephrol.

[31] Shi H, Dong Z, Gao H (2019). LncRNA TUG1 protects against cardiomyocyte ischaemia reperfusion injury by inhibiting HMGB1. Artif Cells Nanomed Biotechnol.

[32] Chen S, Wang M, Yang H, Mao L, He Q, Jin H, Ye ZM, Luo XY, Xia YP (2017). Hu B. LncRNA TUG1 sponges microRNA-9 to promote neurons apoptosis by up-regulated Bcl2l11 under ischemia. Biochem Biophys Res Commun.

[33] Schneider MP, Sullivan JC, Wach PF, Boesen EI, Yamamoto T, Fukai T, Harrison DG, Pollock DM, Pollock JS (2010). Protective role of extracellular superoxide dismutase in renal ischemia/reperfusion injury. Kidney Int.

[34] Dong Q, Lin X, Shen L, Feng Y (2016). The protective effect of herbal polysaccharides on ischemia-reperfusion injury. Int J Biol Macromol.

[35] Kassan A, Pham U, Nguyen Q, Reichelt ME, Cho E, Patel PM, Roth DM, Head BP, Patel HH (2016). Caveolin-3 plays a critical role in autophagy after ischemia-reperfusion. Am J Physiol Cell Physiol.

[36] Kuma A, Matsui M, Mizushima N (2007). LC3, an autophagosome marker, can be incorporated into protein aggregates independent of autophagy: caution in the interpretation of LC3 localization. Autophagy.

[37] Zhang XY, Zhang TT, Song DD, Zhou J, Han R, Qin ZH, Sheng R (2015). Endoplasmic reticulum chaperone GRP78 is involved in autophagy activation induced by ischemic preconditioning in neural cells. Mol Brain.

[38] Chen HH, Mekkaoui C, Cho H, Ngoy S, Marinelli B, Waterman P, Nahrendorf M, Liao R, Josephson L, Sosnovik DE (2013). Fluorescence tomography of rapamycin-induced autophagy and cardioprotection in vivo. Circ Cardiovasc Imaging.

[39] Su Q, Liu Y, Lv XW, Dai RX, Yang XH, Kong BH (2020). LncRNA TUG1 mediates ischemic myocardial injury by targeting miR-132-3p/HDAC3 axis. Am J Physiol Heart Circ Physiol.

[40] Zhang H, Li H, Ge A, Guo E, Liu S, Zhang L (2018). Long non-coding RNA TUG1 inhibits apoptosis and inflammatory response in LPS-treated H9c2 cells by down-regulation of miR-29b. Biomed Pharmacother.

[41] Wang X, Wang R, Bai S, Xiong S, Li Y, Liu M, Zhao Z, Wang Y, Zhao Y, Chen W (2019). Musashi2 contributes to the maintenance of CD44v6 + liver cancer stem cells via notch1 signaling pathway. J Exp Clin Cancer Res.

[42] Kalogeris T, Bao Y, Korthuis RJ (2014). Mitochondrial reactive oxygen species: a double edged sword in ischemia/reperfusion vs preconditioning. Redox Biol.

[43] Yang D, Yu J, Liu HB, Yan XQ, Hu J, Yu Y, Guo J, Yuan Y, Du ZM (2019). The long non-coding RNA TUG1-miR-9a-5p axis contributes to ischemic injuries by promoting cardiomyocyte apoptosis via targeting KLF5. Cell Death Dis.

