# Supplementary figures and images for: Long non-coding RNA TUG1 knockdown promotes autophagy and improves acute renal injury in ischemia-reperfusion-treated rats by binding to microRNA-29 to silence PTEN
Source: BMC Nephrol. 2021 Aug 24;22:288. doi: 10.1186/s12882-021-02473-0 (PMC8385981; doi:10.1186/s12882-021-02473-0)

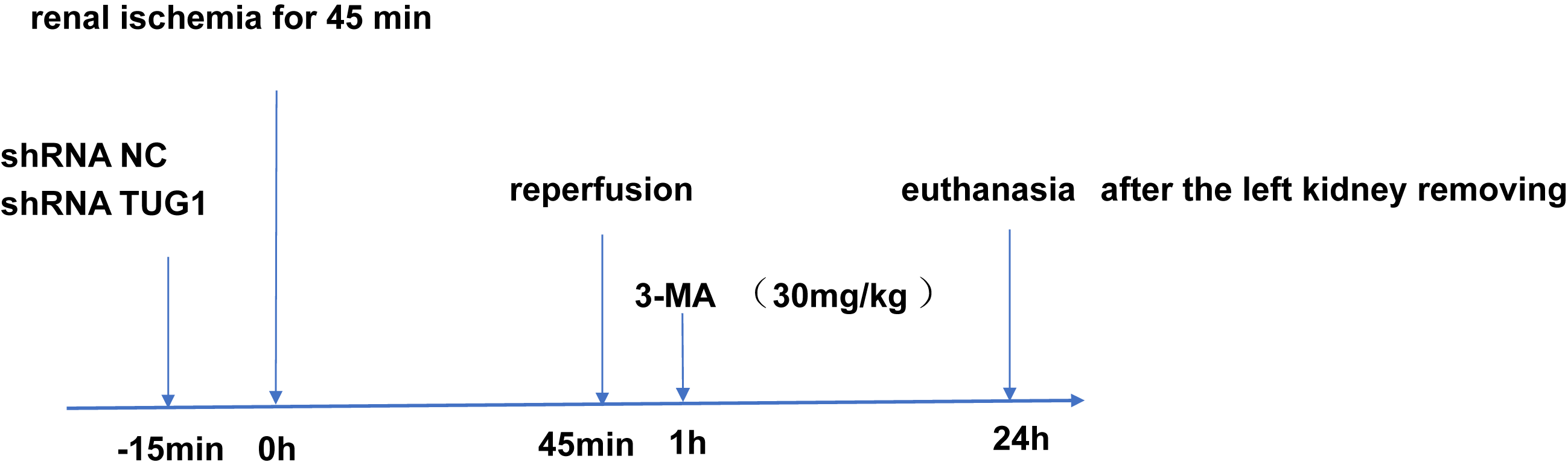

Supplement: Supplementary file 1 — Additional file 1: Supplementary Figures S1, S2 and S3 Flow chart of cell experiments and animal experiments [file 12882_2021_2473_MOESM1_ESM.zip › supplemental figure 1.tiff]

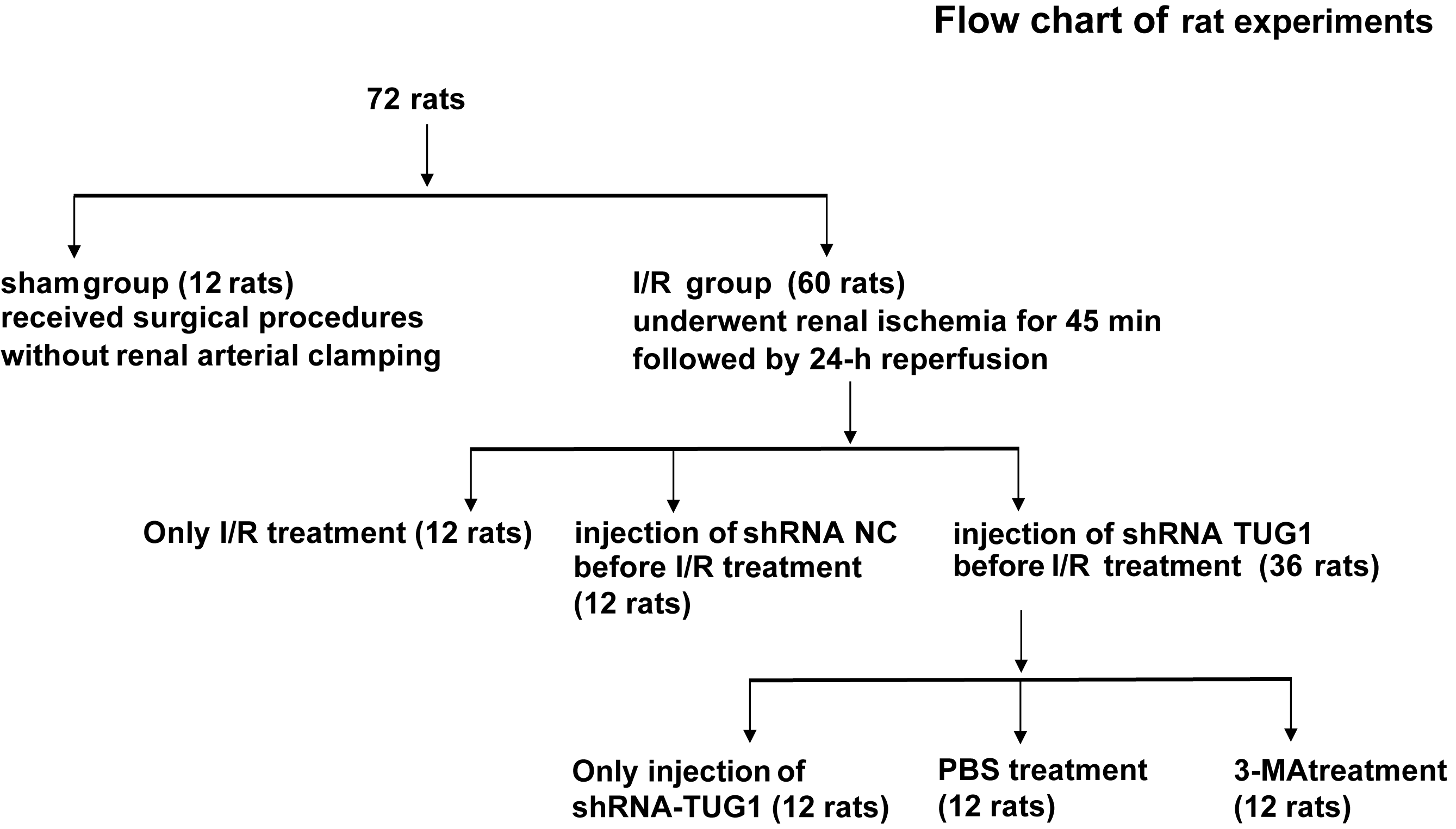

Supplement: Supplementary file 1 — Additional file 1: Supplementary Figures S1, S2 and S3 Flow chart of cell experiments and animal experiments [file 12882_2021_2473_MOESM1_ESM.zip › supplemental figure 2.tiff]

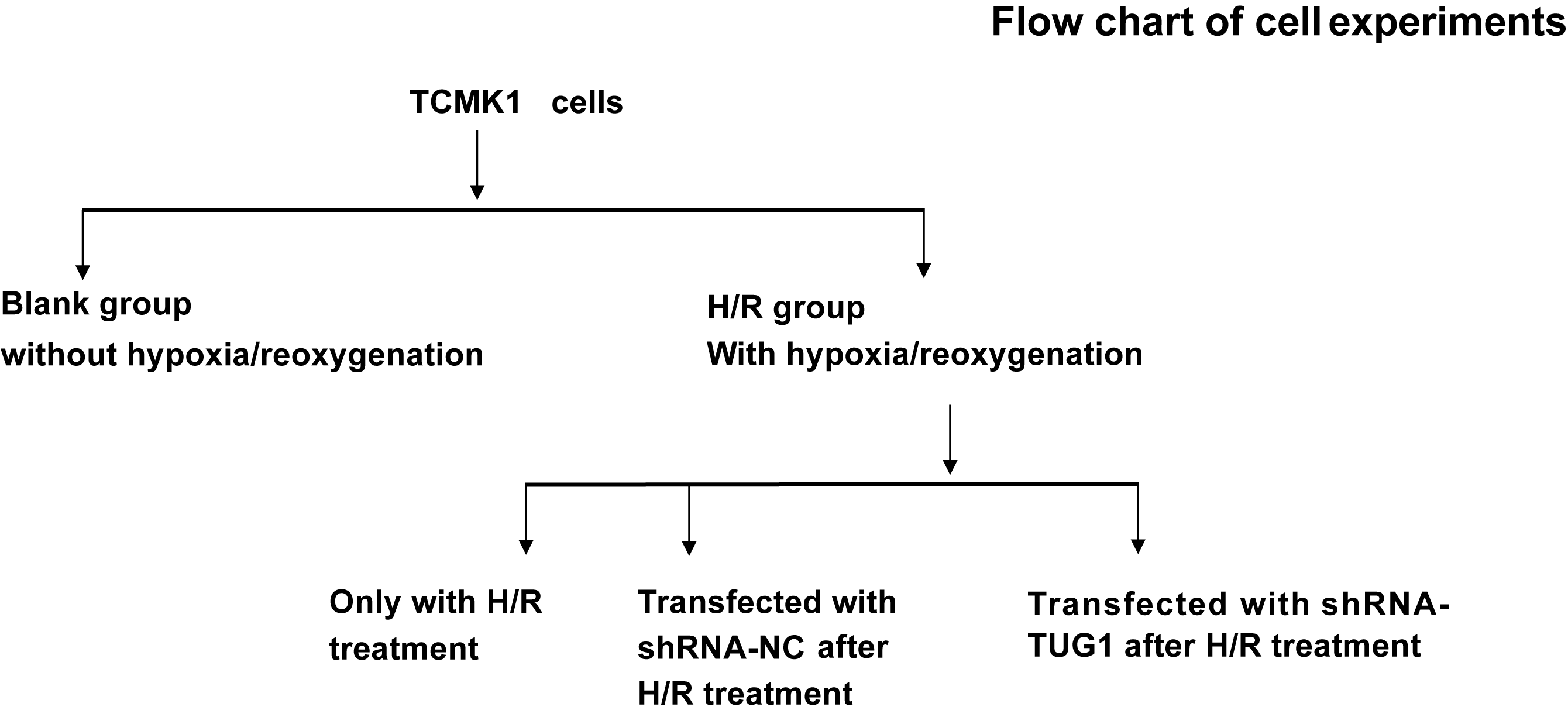

Supplement: Supplementary file 1 — Additional file 1: Supplementary Figures S1, S2 and S3 Flow chart of cell experiments and animal experiments [file 12882_2021_2473_MOESM1_ESM.zip › supplemental figure 3.tiff]
